# The evidence-base for the management of flexor tendon injuries of the hand: Review

**DOI:** 10.1016/j.amsu.2019.10.006

**Published:** 2019-10-15

**Authors:** Athanasius Ishak, Akshaya Rajangam, Ankur Khajuria

**Affiliations:** a Faculty of Life Sciences and Medicine, Centre for Human and Applied Physiological Science (CHAPS), King's College London, Strand, London, WC2R 2LS, United Kingdom; b Department of Plastic Surgery, St Thomas' Hospital, London, UK; c Kellogg College, University of Oxford, Oxford, UK

**Keywords:** Hand surgery, Primary flexor tendon repair, Wide awake surgery, Rehabilitation, Patient reported outcome measures

## Abstract

• There is no consensus on the optimal flexor tendon repair technique at each anatomical flexor zone.
• There is paucity of high quality evidence.
• Heterogenous study designs limit inter-study comparisons.
• Patient reported outcome measures are crucial but there is a perennial need for robust disease-specific tools to be utilised.

## 1 Introduction

Hand injuries account for up to 20% of all presentations to emergency departments and cost the National Health Service (NHS) over £100 million per year [1]. Flexor tendon injuries are common and may have debilitating sequalae, with re-operation rates as high as 11% [2], culminating in poor patient-reported outcomes [3]. Early active mobilisation (EAM) protocols are commonly used for post-operative rehabilitation, however, there is no definitive consensus on the ideal rehabilitation regimen. “Place and hold” regimes are also popular and although they contain an active component are not considered EAM. There is no consensus on the ideal flexor tendon repair (FTR) technique. Numerous studies have evaluated the merits of various suture configurations, however, directly comparing such studies is difficult due to significant methodological heterogeneity. Consequently, there may be variability in management between units and suboptimal adherence to best practice [1]. Although the results of ex vivo biomechanical studies correlate with the in vivo biomechanical properties of sutured flexor tendons, the focus of this review will be to summarise the clinical evidence base for primary adult FTR techniques at each anatomical zone to provide a clear overview for the reader and suggestions for future work.

## 1.1 Flexor tendon zones of the hand

**Fig. 1:**
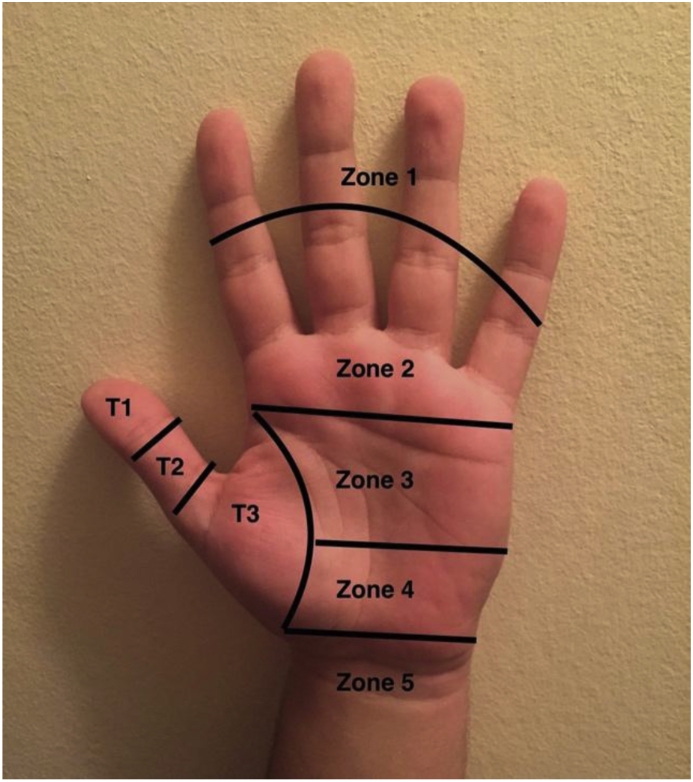
The anatomical zones of tendon injuries (Verdan). T1 = thumb zone 1, T2 = thumb zone 2, T3 = thumb zone 3.

The flexor tendons are split into five zones based on the Verdan classification (Fig. 1) [4].

## 1.2 Core and epitendinous sutures

The aim of FTR is to achieve a balance between repair strength and tendon glide. The ideal characteristics of a primary FTR have been outlined [5].

• Sutures easily and securely placed in the tendon
• Smooth juncture of tendon ends
• Minimal gapping at the repair site (less than 3 mm) [6].
• Minimal interference with tendon vascularity
• Sufficient strength to permit EAM

The initial strength of a repaired tendon depends on the number of suture strands crossing the repair site, core suture purchase length, anchoring technique, lock diameter and core suture material [7]. Factors such as trauma (from injury and surgery), tendon ischaemia, tendon immobilisation and repair site gapping induce adhesion formation [5].

Epitendinous sutures can improve the strength of repairs by 10–50% [5]. They impart three major benefits: improved strength, minimal gapping and a smooth glide [8]. Many hand surgeons favour an epitenon-first approach to reduce the bulk of the repair site and minimize the risk of triggering. Continuous epitendinous sutures should be placed circumferentially 2 mm from the repair site to achieve the strongest repair [9]. The strength of the repair can be enhanced by maintaining equal tension across all suture strands [10], minimising the number of knots needed, locating them away from the tendon repair site [11] and by dorsally placing core sutures [12].

## 1.3 Wide awake FTR

Local lidocaine and epinephrine can be used for finger and hand anaesthesia. This enables intra-operative testing of the tendon which improves outcomes and reduces post-operative rupture and tenolysis [13,14]. However, no studies have directly compared the results of surgery performed under local anaesthetic with regional or general anaesthesia.

## 1.4 Zone 1 repair

Zone 1A injuries may result in a distal FDP tendon stump that is less than 1 cm long necessitating a tendon to bone repair. Such injuries are associated with a complication rate of up to 60% [15]. The commonest repair technique used is the ‘‘button-over-nail’’ repair. However, this is associated with limited range of motion at the distal interphalangeal joint (DIPJ) which significantly reduces the functionality of the digit and patient satisfaction [16]. Furthermore, as sutures project externally there is a significant infection risk and snagging of the button and rupture of the repair have been reported [17]. The “Shepherd's Crook” repair is a simple variation using a k-wire as an external strut instead of the classic button [18]. This is particularly useful in situations where a *trans*-articular k-wire is to be used anyway and in situations requiring temporary joint immobilisation, but these are not pre-requisite. The bent k-wire dynamically maintains tension and apposition of tendon to bone which may reduce gapping. It also has the benefit of avoiding skin pressure necrosis and damage to the nail complex. Nevertheless, there are clear drawbacks such as the risk of infection, k-wire prominence and difficulties with patient compliance. Importantly, robust clinical data about outcomes and complications for FTR are lacking and some have voiced concerns about a theoretical loss of suture tension [19].

The use of micro bone suture anchors is an alternative that may avoid the morbidity associated with pull-out suture repairs. Multiple anchors can be placed which enhances strength and allows for secure EAM culminating in shorter recovery times than with a button technique - 9.77 ± 2.01 weeks vs 12.23 ± 3.68 weeks [20]. Huq et al., 2013 reported 77% of patients achieved DIPJ range of motion greater than 40° and 56% of patients achieved good or excellent DIPJ range of motion (modified Strickland criteria) [21] compared with 66% and 38% respectively when using a modified button-over nail technique (Schaller et al., 2010) [22]. The strength of the suture anchor material correlates with the force to failure and this ought to influence material selection [23]. The main disadvantage of bone anchors is cost although this may be offset by lower complication rates, better functional outcomes and shorter recovery times. The use of suture anchors may be contraindicated in patients older than 75 years because of poor bone quality [23], although there are no clinical reports of osteoporosis affecting bone anchor repairs and zone 1 injuries primarily affect younger patients [24].

## 1.5 Zone 2 repair

Referred to as “no man's land”, repair here is challenging because tendon glide must be restored within a tight fibro-osseous sheath. Lacerations in zone 2 may involve the FDS and FDP tendons and both should be repaired with extra care taken not to disrupt campers chiasm [25]. If repair of both slips of the FDS results in a bulky tendon which impedes glide then it is acceptable for one slip to be resected [26].

Tendons can be safely exposed by using a Bruner's incision or a mid-lateral Bunnell's incision [25]. The latter approach is preferred as it avoids narrow flap tips, permits wider exposure and places intact soft tissue directly over the tendons [26]. It is important to preserve the A2 and A4 pulleys to prevent bowstringing although the A2 can be partially vented if necessary, but this ought to be done meticulously as venting has been associated with increased glide resistance and reduced finger range of motion [27]. Experimentally, the addition of biological lubricants after FTR may improve functional outcomes although there is no robust clinical evidence for this [28].

### 1.5.1 Two-strand repairs

Two-strand repairs have largely fallen out of favour due to very high rupture rates [29] and in a survey (2018) 75.9% of surgeons stated they performed suture repair with at least four strands [30]. More recently, Georgescu et al., 2019 reported no post-operative ruptures using their modified Brunelli pull-out technique [31]. Their repair does not leave any knots on or inside the tendon and moves the point of maximum tension to the tendon insertion site thus avoiding tension between tendon stumps and a bulky repair. Moreover, as each muscular contracture results in tighter contact between tendon stumps the risk of gap formation is minimised. This repair has only been used in 58 patients but achieved excellent results in all cases (Strickland criteria), yet 31% of patients had extension deficits of 10–20° and a complete range of flexion was restored in 57.5% of fingers [31,32].

### 1.5.2 Four-strand repairs

The four-strand repair is the most commonly used yet there are few published clinical studies evaluating the outcomes in zone 2 [30]. In one study using a 4-strand repair only one rupture was reported (2.5%) but this occurred because the patient did not comply with the rehabilitation protocol [33]. The surgeons used a variety of four strand repairs including the two Tajima or modified Kessler sutures or a modified Kessler suture and mattress or locking mattress suture using 3–0 or 4-0 braided synthetic material. A simple running epitendinous repair of 6–0 polypropylene was also used. The outcomes from each type of repair are not reported individually but overall in zone 2, 95% of fingers had excellent-good functionality (53% and 42% respectively, Strickland-Glogovac criteria) [33]. More recently a 4-strand modified Kessler core suture (4–0 PDS) with an epitendinous locking suture (6-0 Nylon) technique has been described which achieved rupture rates of only 2.3% and excellent-good Strickland scores in 91.4% of fingers [34].

Silfverskiöld et al., 1994 evaluated a cross-lock cruciate repair in 46 patients (55 digits) [35]. Only two ruptures were reported and the mean active DIPJ and PIPJ range of motion at 6 months were 63° and 94° respectively. The cross-lock repair can be completed with a single suture which minimises the bulk of the repair and makes it technically easier than many other repairs. Disadvantages include exposed suture on the surface of the tendon, increased tissue handling, and that additional tensioning of the repair cannot be easily achieved at the time of final knot tying [36].

### 1.5.3 Six-strand repairs

Osada et al., 2006 reported excellent outcomes for zone 2 FTR using the Yoshizu 1 technique (Y1) or a triple-looped suture technique [29]. No ruptures occurred and 96% of patients achieved excellent functional outcomes (Strickland-Glogovac criteria). No significant differences were found between patients who underwent either six-strand repair. The Y1 technique is a combination of the Tsuge suture with a looped thread and the modified Kessler suture using a double strand with two needles. The triple-looped technique uses three Tsuge sutures with a 4-0 looped thread. Patients remained in hospital for four weeks after surgery for supervised rehabilitation which may explain why other studies evaluating the Y1 technique reported a 5.1% rupture rate and only 82% good-excellent functional outcomes [37]. The clinical outcomes of the Y1 technique in each of the subzones of zone 2 have been assessed over 102 tendon repairs [38]. Good-excellent function was restored to approximately 83% of tendons yet overall patients with zone 2C lacerations tended to fare worse and four ruptures were reported all occurring in digits with zone 2B injuries [38].

Zone 2C is the most difficult area to obtain satisfactory active digital motion as it represents the area underneath the A2 pulley where tendons are the most confined. The optimum approach if both the FDP and FDS have been lacerated here is controversial although repair of the FDP alone and releasing the A2 pulley have been recommended [39,40].

The Lim-Tsai six-strand double-loop technique has been shown to restore excellent function in 78% of patients (Strickland-Glogovac criteria) and compared to two-strand repairs it was associated with greater total active motion, fewer complications and faster recovery [41]. In another report, the use of Lim-Tsai sutures with place and hold exercises achieved 81% good-excellent results (revised Strickland criteria) [42]. Generally, the Lim-Tsai repair is recognised for its superior strength, however, it is limited by the need for an intra-tendinous knot and increased tissue handling [36]. A modified six-strand double loop technique has also been shown to achieve 81% excellent-good outcomes (Strickland classification) and a rupture rate of only 1.9% [43]. The technique described by the authors of this study is relatively simple and faster to perform than some other six-strand configurations [43]. Nevertheless, there are clear shortcomings; ruptures rates are higher than with the Y1 and triple-looped techniques and tendon healing may be disturbed by multiple intra-tendinous knots [44].

### 1.5.4 Eight-strand & ten-strand repairs

Eight-strand and ten-strand repairs are complex, time consuming, bulky and require increased tissue handling which limits their role in routine practise.

### 1.5.5 Teno Fix device

The Teno Fix device was developed for zone 2 repairs based on the premise that the ideal repair was easy to apply and reproducible. A randomised blinded clinical trial comparing the Teno Fix device to a locked four strand cruciate repair reported no ruptures using the Teno Fix device but an 18% rupture rate in the control group [45]. No significant differences in functional outcomes were found. A key advantage of the device is its use of a knotless anchor as post-operative tendon rupture usually occurs at the site of suture knots [[46], [47], [48]].

## 1.6 Zone 3 repair

Zone 3 injuries are relatively rare and tend to be open injuries although closed rupture injuries have been reported [49]. Al-Qattan et al., 2011 achieved excellent outcomes in 38/40 fingers and no ruptures using a two and three ‘figure of eight’ core suture configuration in combination with a continuous epitendinous suture to repair flexor superficialis and profundus tendon lacerations [50]. Suture knots were not buried, and the authors deliberately did not repair damaged lumbricals to avoid fibrosis and deformity. Ultimately, eighteen fingers had mild flexion contractures of the PIP joint (5–20°) and two had moderate contractures (35° and 40°). Generally, a good prognosis with safe EAM can be expected especially if there is no concurrent neurovascular compromise due to the anatomically favourable characteristics of zone 3 [51,52].

## 1.7 Zone 4 repair

Pure tendon injuries are rare in Zone 4, due to protection from the flexor retinaculum. The context of tendon repairs in this zone is usually multiple tendon injuries and neurovascular compromise. A review has concluded that the typical management includes direct tendon repair following the release of the transverse carpal ligament [53]. In one case report, full range of motion was successfully restored following a closed spontaneous FDP rupture using a four-strand core Adelaide repair with a running epitendinous suture [54].

## 1.8 Zone 5 repair

Despite being a common site for flexor tendon injury, there are few reports on the outcomes of primary tendon repair in Zone 5 injuries [55,56]. Zone 5 injuries are often associated with concurrent neurovascular compromise which requires surgical intervention and impacts rehabilitation [53]. Nasab et al., 2013 found the modified Kessler technique restored excellent-good results (Buck-Gramko score) in 75% of patients [56]. Similarly, Bal et al., 2011 reported excellent-good results for total active motion in 83% of digits and recovery of grip strength to an average of 53% of the uninjured hand [55]. Furthermore, using their repair technique they showed that zone 5 injuries had better anatomic improvement than injuries in zone 2 and lower re-operation rates [55]. Good functional and technical outcomes using the Kessler repair have also been reported by Raza et al., 2014 although these results are limited by a lack of standardisation of rehabilitation protocol [57].

## 1.9 Thumb injuries

5.6% of all acute tendon ruptures in the hand and wrist involve the flexor pollicis longus (FPL) and concomitant neurovascular injury has been reported in 82% of cases [58,59]. The FPL tendon often retracts after division and a region of relative avascularity within zone 2 is recognised which complicates repair and contributes to the significant post-operative rupture rate [60,61].

Using a six-strand M-Tang repair, Pan et al., 2017 achieved excellent results with minimal deficits in interphalangeal joint extension (on average 13° in 14 thumbs) in 45% of patients with zone 2 lacerations [62]. No ruptures were reported, and the authors attributed this to venting at least one of the A1 or A2 pulleys. Giesen et al., 2009 also reported no tendon ruptures using the Tang technique of 3 Tsuge sutures and 82% excellent-good results (Buck-Gramcko assessment) [61]. An end-to-end repair has also been used and excellent-good outcomes were attained in 73.3% and 88.8% of zone 2 and 3 injuries respectively (Buck-Gramcko score); importantly, this technique could restore optimal pinch strength despite neurovascular injury [63].

Other repair techniques such as the motion-stable Mantero technique and the Kessler four-strand repair with a Silfverskiöld circumferential suture have resulted in inadequate interphalangeal joint mobility and poor overall functional results or have been deemed impractical for routine use respectively [60,64].

## 2 Advanced methods of FTR

Typically, patients who present more than 1month after trauma are not eligible for direct repair, however, Tang JB. 2013 has reported positive outcomes using late direct repair in patients for whom direct approximation of the tendon ends is possible [65]. Tendon tension is markedly greater during such procedures and the use of at least a 6-strand core suture repair is recommended. Z-plasty lengthening of flexor tendons is a useful adjunct to help compensate for the loss of elasticity of muscle fibres [65,66]. This ought to be considered only after taking into account hand function and patient compliance with rehabilitation. There are no preoperative guidelines to determine suitability for late direct repair and judgement must be made on a single digit basis intraoperatively. Ultimately, the surgeon's level of expertise will impact their ability to make informed judgements in such scenarios which fall outside the realm of guidelines.

### 2.1 Patient reported outcome measures (PROMs)

Assessing clinical outcomes is necessary but insufficient for a complete assessment of patient care [67]. PROMs are increasingly being used to evaluate healthcare services as they enable an assessment of the value of an intervention and capture the patient's response [68,69]. A robust PRO tool must be reliable, valid (content validity, construct validity and responsiveness), sensitive, easy to interpret and acceptable to the patient and investigator [69,70]. The most frequently used tools for measuring PROMs in hand surgery are the Disabilities of the Arm, Shoulder and Hand (DASH) questionnaire and the Michigan Hand Outcomes Questionnaire (MHQ) [67,[71], [72], [73]].

A systematic review by Wormald et al., 2019 reported that the psychometric properties of the most commonly used PROMS in hand surgery are not adequately described [67]. The available PROMs vary in their scope – some are disease specific while others are domain specific and if used in isolation may miss important information [74]. Furthermore, the evidence-base comparing the various PROMs against each other and against flexor tendon zones is lacking. Rodrigues et al., 2018 suggested PROMs should be used in conjunction with other clinical measurements and this is a step in the right direction [75]. Future efforts ought to focus on developing and clinically appraising robust and sensitive tools to evaluate hand conditions post-FTR surgery.

## 2.2 Conclusion

Paucity of high-quality evidence renders it difficult to establish the most optimal FTR technique for each anatomical zone. The majority of published studies are cadaveric/animal studies whilst the methodological heterogeneity of clinical studies limits direct comparison. As a such, it is of paramount importance that data from experimental studies are interpreted properly in the context of their limitations. Informative findings which all authors should strive to report on include the training and expertise of the surgeon performing FTR as well as the percentage of pulley venting (if applicable). Long-term follow up with the view of accurately recording compensatory mechanisms which may contribute to a satisfactory result after surgery at the expense of function is essential. More robust longitudinal prospective studies with larger sample sizes are needed with the incorporation of robust PROMs tools for comprehensive outcome assessment and establishing best practice.

## Ethical approval

Not applicable

## Sources of funding

There are no sources of funding to declare.

## Author contribution

Study concept: Ankur Khajuria & Athanasius Ishak.

Data analysis and interpretation: Athanasius Ishak & Akshaya Rajangam.

Writing the paper: Athanasius Ishak, Akshaya Rajangam and Ankur Khajuria.

## Guarantor

Athanasius Ishak.

### Data statement

Not applicable.

## Provenance and peer review

Not commissioned, externally peer reviewed.

## Consent

Not applicable.

## Declaration of competing interest

None of the authors have any conflicts of interests to report.

## References

[1] Rudge W.B.J., James M. (2014). Flexor tendon injuries in the hand: a UK survey of repair techniques and suture materials—are we following the evidence?. ISRN Plast. Surg..

[2] Wong J.K.F., Peck F. (2014). Improving results of flexor tendon repair and rehabilitation. Plast. Reconstr. Surg..

[3] Sammer D.M., Chung K.C. (2014). Advances in the healing of flexor tendon injuries. Wound Repair Regen..

[4] VERDAN C.E. (1960). Primary repair of flexor tendons. J. Bone Joint Surg. Am..

[5] Strickland J.W. (2005). The scientific basis for advances in flexor tendon surgery. J. Hand Ther..

[6] Gelberman R.H., Boyer M.I., Brodt M.D., Winters S.C., Silva M.J. (1999). The effect of gap formation at the repair site on the strength and excursion of intrasynovial flexor tendons. An experimental study on the early stages of tendon-healing in dogs. J. Bone Joint Surg. Am..

[7] Yoneda S., Okubo H., Linderman S.W., Kusano N., Silva M.J., Thomopoulos S., Kanaya F., Gelberman R.H. (2018). The effect of modified locking methods and suture materials on Zone II flexor tendon repair—an ex vivo study. PLoS One.

[8] Galvez M.G., Comer G.C., Chattopadhyay A., Long C., Behn A.W., Chang J. (2017). Gliding resistance after epitendinous-first repair of flexor digitorum profundus in zone II. J. Hand Surg. Am..

[9] Azar Frederick M., Canale S. Terry, Beaty James H., Campbell Willis C., Frederick S.T.C., Azar M., Beaty James H. (2017). Flexor and extensor tendon injuries. Campbell's Oper. Orthpaedics.

[10] Trail I.A., Powell E.S., Noble J. (1989). An evaluation of suture materials used in tendon surgery. J. Hand Surg. Br..

[11] Aoki M., Pruitt D.L., Kubota H., Manske P.R. (1995). Effect of suture knots on tensile strength of repaired canine flexor tendons. J. Hand Surg. Br..

[12] Soejima O., Diao E., Lotz J.C., Hariharan J.S. (1995). Comparative mechanical analysis of dorsal versus palmar placement of core suture for flexor tendon repairs. J. Hand Surg. Am..

[13] Lalonde D.H., Martin A.L. (2013). Wide-awake flexor tendon repair and early tendon mobilization in zones 1 and 2. Hand Clin..

[14] Tang J.B. (2015). Wide-Awake primary flexor tendon repair, tenolysis, and tendon transfer. Clin. Orthop. Surg..

[15] Evans R.B. (1990). A study of the Zone I flexor tendon injury and implications for treatment. J. Hand Ther..

[16] Bidwai A.S.C., Feldberg L. (2012). The button-over-nail technique for zone I flexor tendon injuries. Hand Surg..

[17] Kang N., Marsh D., Dewar D. (2008). The morbidity of the button-over-nail technique for zone 1 flexor tendon repairs. Should we still be using this technique?. J. Hand Surg. Eur..

[18] De Spirito D., Giunchi D. (2017). The pull-out K-wire anchorage: the “Shepherd's Crook” technique. Tech. Hand Up. Extrem. Surg..

[19] Karslioglu B., Tekin A.C., Tasatan E. (2018). The weakest point of “the Shepherd's Crook” technique. Tech. Hand Up. Extrem. Surg..

[20] McCallister W.V., Ambrose H.C., Katolik L.I., Trumble T.E. (2006). Comparison of pullout button versus suture anchor for zone I flexor tendon repair. J. Hand Surg. Am..

[21] Huq S., George S., Boyce D.E. (2013). The outcomes of zone 1 flexor tendon injuries treated using micro bone suture anchors. J. Hand Surg. Eur..

[22] Schaller P., Baer W. (2010). Motion-stable flexor tendon repair with the Mantero technique in the distal part of the fingers. J. Hand Surg. (European).

[23] Matsuzaki H., Zaegel M.A., Gelberman R.H., Silva M.J. (2008). Effect of suture material and bone quality on the mechanical properties of zone I flexor tendon–bone reattachment with bone anchors. J. Hand Surg. Am..

[24] Huq S., George S., Boyce D.E. (2013). Zone 1 flexor tendon injuries: a review of the current treatment options for acute injuries. J. Plast. Reconstr. Aesthet. Surg..

[25] Griffin M. (2012). An overview of the management of flexor tendon injuries. Open Orthop. J..

[26] Lutsky K.F., Giang E.L., Matzon J.L. (2015). Flexor tendon injury, repair and rehabilitation, orthop. Clin. North Am..

[27] Tanaka T., Amadio P.C., Zhao C., Zobitz M.E., An K.-N. (2004). The effect of partial A2 pulley excision on gliding resistance and pulley strength in vitro. J. Hand Surg. Am..

[28] Zhao C., Wei Z., Reisdorf R.L., Thoreson A.R., Jay G.D., Moran S.L., An K.-N., Amadio P.C. (2014). The effects of biological lubricating molecules on flexor tendon reconstruction in a canine allograft model in vivo. Plast. Reconstr. Surg..

[29] Osada D., Fujita S., Tamai K., Yamaguchi T., Iwamoto A., Saotome K. (2006). Flexor tendon repair in zone II with 6-strand techniques and early active mobilization. J. Hand Surg. Am..

[30] Bigorre N., Delaquaize F., Degez F., Celerier S. (2018). Primary flexor tendons repair in zone 2: current trends with GEMMSOR survey results. Hand Surg. Rehabil..

[31] Georgescu A.V., Matei I.R., Olariu O. (2019). Zone II flexor tendon repair by modified Brunelli pullout technique and very early active mobilization. J. Hand Surg. Am..

[32] Georgescu A.V., Matei I.R., Capota I.M., Ardelean F., Olariu O.D. (2011). Modified Brunelli pull-out technique in flexor tendon repair for zone II: a study on 58 cases. HAND.

[33] Klein L. (2003). Early active motion flexor tendon protocol using One splint. J. Hand Ther..

[34] Güntürk Ö.B., Kayalar M., Kaplan İ., Uludağ A., Özaksar K., Keleşoğlu B. (2018). Results of 4-strand modified Kessler core suture and epitendinous interlocking suture followed by modified Kleinert protocol for flexor tendon repairs in Zone 2. Acta Orthop. Traumatol. Turcica.

[35] Silfverskiöld K.L., May E.J. (1994). Flexor tendon repair in zone II with a new suture technique and an early mobilization program combining passive and active flexion. J. Hand Surg. Am..

[36] Chauhan A., Palmer B.A., Merrell G.A. (2014). Flexor tendon repairs: techniques, eponyms, and evidence. J. Hand Surg. Am..

[37] Moriya K., Yoshizu T., Maki Y., Tsubokawa N., Narisawa H., Endo N. (2015). Clinical outcomes of early active mobilization following flexor tendon repair using the six-strand technique: short- and long-term evaluations. J. Hand Surg. (European).

[38] Moriya K., Yoshizu T., Tsubokawa N., Narisawa H., Matsuzawa S., Maki Y. (2017). Outcomes of flexor tendon repairs in zone 2 subzones with early active mobilization. J. Hand Surg. Eur..

[39] Tang J.B. (1994). Flexor tendon repair in zone 2C. J. Hand Surg. Am..

[40] Moriya K., Yoshizu T., Tsubokawa N., Narisawa H., Hara K., Maki Y. (2016). Clinical results of releasing the entire A2 pulley after flexor tendon repair in zone 2C. J. Hand Surg. Am..

[41] Hoffmann G.L., Büchler U., Vögelin E. (2008). Clinical results of flexor tendon repair in zone II using a six-strand double-loop technique compared with a two-strand technique. J. Hand Surg. (European).

[42] Lim B., Tsai T. (1996). The six-strand technique for flexor tendon repair. Atlas Hand. Clin..

[43] Savvidou C., Tsai T.M. (2015). Clinical results of flexor tendon repair in zone II using a six strand double loop technique. J Hand Microsurg.

[44] Savage R., Risitano G. (1989). Flexor tendon repair using a “‘SIX strand’” method OF repair and early active mobilisation. J. Hand Surg. (British).

[45] Su B.W., Solomons M., Barrow A., Senoge M.E., Gilberti M., Lubbers L., Diao E., Quitkin H.M., Rosenwasser M.P. (2005). Device for zone-II flexor tendon repair. J. Bone Jt. Surg..

[46] WONG J.K.F., CEROVAC S., FERGUSON M.W.J., MCGROUTHER D.A. (2006). The cellular effect of a single interrupted suture on tendon. J. Hand Surg. Am..

[47] Rocchi L., Merolli A., Genzini A., Merendi G., Catalano F. (2008). Flexor tendon injuries of the hand treated with TenoFix: mid-term results. J. Orthop. Traumatol..

[48] Trail I.A., Powell E.S., Noble J. (1992). The mechanical strength of various suture techniques. J. Hand Surg. Br. Eur..

[49] Ostric S.A., Russell R.C., Petrungaro J. (2010). Closed zone III rupture of the flexor digitorum profundus tendons of the right index, long, and ring fingers in a bowler: gutterball Syndrome. Hand.

[50] Al-Qattan M.M. (2011). Flexor tendon repair in zone III. J. Hand Surg. Eur..

[51] Athwal G.S., Wolfe S.W. (2005). Treatment of acute flexor tendon injury: zones III–V. Hand Clin..

[52] Mehling I.M., Arsalan-Werner A., Sauerbier M. (2014). Evidence-based flexor tendon repair. Clin. Plast. Surg..

[53] Klifto C.S., Capo J.T., Sapienza A., Yang S.S., Paksima N. (2018). Flexor tendon injuries. J. Am. Acad. Orthop. Surg..

[54] Whitehouse H., Chan J.C.Y., James M. (2018). Spontaneous closed zone IV rupture of flexor digitorum profundus tendon to the fifth finger. Case Reports Plast. Surg. Hand Surg..

[55] Bal S., Oz B., Gurgan A., Memis A., Demirdover C., Sahin B., Oztan Y. (2011). Anatomic and functional improvements achieved by rehabilitation in Zone II and Zone V flexor tendon injuries. Am. J. Phys. Med. Rehabil..

[56] Mehdi Nasab S.A., Sarrafan N., Saeidian S.R., Emami H. (2013). Functional outcome of flexor tendon repair of the hand at Zone 5 and post operative early mobilization of the fingers. Pakistan J. Med. Sci..

[57] Raza M.S., Jaffery S.A.Y., Khan F.A. (2014). Flexor Zone 5 cut injuries: emergency management and outcome. J. Coll. Physicians Surg. Pak..

[58] Nunley J.A., Levin L.S., Devito D., Goldner R.D., Urbaniak J.R. (1992). Direct end-to-end repair of flexor pollicis longus tendon lacerations. J. Hand Surg. Am..

[59] de Jong J.P., Nguyen J.T., Sonnema A.J.M., Nguyen E.C., Amadio P.C., Moran S.L. (2014). The incidence of acute traumatic tendon injuries in the hand and wrist: a 10-year population-based study. Clin. Orthop. Surg..

[60] Sirotakova M., Elliot D. (2004). Early active mobilization of primary repairs of the flexor pollicis longus tendon with two Kessler two-strand core sutures and a strengthened circumferential suture. J. Hand Surg. Br..

[61] GIESEN T., SIROTAKOVA M., COPSEY A.J., ELLIOT D. (2009). Flexor pollicis longus primary repair: further experience with the tang technique and controlled active mobilization. J. Hand Surg. (European).

[62] Pan Z.J., Qin J., Zhou X., Chen J. (2017). Robust thumb flexor tendon repairs with a six-strand M-Tang method, pulley venting, and early active motion. J. Hand Surg. (European).

[63] Oztürk K., Orhun E., Polatkan O., Polatkan S. (2004). [Long-term results of early primary repair of flexor pollicis longus tendon injuries]. Acta Orthop. Traumatol. Turcica.

[64] Schaller P. (2010). Repair of the flexor pollicis longus tendon with the motion-stable Mantero technique. J. Plast. Surg. Hand Surg..

[65] Tang J. (2013). Uncommon methods of flexor tendon and tendon-bone repairs and grafting. Hand Clin..

[66] Le Viet D. (1986). Flexor tendon lengthening by tenotomy at the musculotendinous junction. Ann. Plast. Surg..

[67] Wormald J.C.R., Geoghegan L., Sierakowski K., Price A., Peters M., Jain A., Rodrigues J.N. (2019). Site-specific patient-reported outcome measures for hand conditions. Plast. Reconstr. Surg. - Glob. Open..

[68] Cohen W.A., Mundy L.R., Ballard T.N.S., Klassen A., Cano S.J., Browne J., Pusic A.L. (2016). The BREAST-Q in surgical research: a review of the literature 2009–2015. J. Plast. Reconstr. Aesthet. Surg..

[69] Pusic A.L., Lemaine V., Klassen A.F., Scott A.M., Cano S.J. (2011). Patient-reported outcome measures in plastic surgery: use and interpretation in evidence-based medicine. Plast. Reconstr. Surg..

[70] Reeve B.B., Wyrwich K.W., Wu A.W., Velikova G., Terwee C.B., Snyder C.F., Schwartz C., Revicki D.A., Moinpour C.M., McLeod L.D., Lyons J.C., Lenderking W.R., Hinds P.S., Hays R.D., Greenhalgh J., Gershon R., Feeny D., Fayers P.M., Cella D., Brundage M., Ahmed S., Aaronson N.K., Butt Z. (2013). ISOQOL recommends minimum standards for patient-reported outcome measures used in patient-centered outcomes and comparative effectiveness research. Qual. Life Res..

[71] SooHoo N.F., McDonald A.P., Seiler J.G., McGillivary G.R. (2002). Evaluation of the construct validity of the DASH questionnaire by correlation to the SF-36. J. Hand Surg. Am..

[72] Offenbächer M., Ewert T., Sangha O., Stucki G. (2003). Validation of a German version of the “disabilities of arm, shoulder and hand” questionnaire (DASH-G). Z. Rheumatol..

[73] Atroshi I., Gummesson C., Andersson B., Dahlgren E., Johansson A. (2000). The disabilities of the arm, shoulder and hand (DASH) outcome questionnaire: reliability and validity of the Swedish version evaluated in 176 patients. Acta Orthop. Scand..

[74] Wormald J.C.R., Rodrigues J.N. (2018). Outcome measurement in plastic surgery. J. Plast. Reconstr. Aesthet. Surg..

[75] Rodrigues J.N., Neblett C. (2018). How to use patient-reported outcome measures with other clinical measurements in clinical reports. J. Hand Surg. (European).

